# Moracin C, A Phenolic Compound Isolated from *Artocarpus heterophyllus*, Suppresses Lipopolysaccharide-Activated Inflammatory Responses in Murine Raw264.7 Macrophages

**DOI:** 10.3390/ijms17081199

**Published:** 2016-07-25

**Authors:** Xue Yao, Dang Wu, Ningning Dong, Ping Ouyang, Jiaqian Pu, Qian Hu, Jingyuan Wang, Weiqiang Lu, Jin Huang

**Affiliations:** 1 Shanghai Key Laboratory of New Drug Design, School of Pharmacy, East China University of Science and Technology, 130 Mei Long Road, Shanghai 200237, China; shirleyyao715@gmail.com (X.Y.); wudang19891013@163.com (D.W.); zoednn@126.com (N.D.); ouyangping2012@yeah.net (P.O.); pujiaqian2014@163.com (J.P.); huqian_ECUST@163.com (Q.H.); joan_wangjy@outlook.com (J.W.); 2 Shanghai Key Laboratory of Regulatory Biology, Institute of Biomedical Sciences and School of Life Sciences, East China Normal University, 500 Dongchuan Road, Shanghai 200241, China

**Keywords:** inflammation, MAPKs, Moracin C, NF-κB, phenols

## Abstract

*Artocarpus heterophyllus*, a popular tropical fruit commonly known as the jackfruit tree, is normally planted in subtropical or tropical areas. Since a variety of phytochemicals isolated from *A. heterophyllus* have been found to possess potently anti-inflammatory, antiviral and antimalarial activities, researchers have devoted much interest to its potential pharmaceutical value. However, the exact mechanism underlying its anti-inflammatory activity is not well characterized. In this study, seven natural products isolated from *A. heterophyllus*, including 25-Hydroxycycloart-23-en-3-one (**HY**), Artocarpin (**AR**), Dadahol A (**DA**), Morachalcone A (**MA**), Artoheterophyllin B (**AB**), Cycloheterophyllin (**CY**) and Moracin C (**MC**) were collected. Lipopolysaccharide (LPS)-stimulated inflammatory response in RAW264.7 macrophages were used in this study. Among these compounds, **MC** significantly inhibited LPS-activated reactive oxygen species (ROS) and nitric oxide (NO) release without marked cytotoxicity. Furthermore, **MC** effectively reduced LPS stimulated up-regulation of mRNA and protein expression of inducible nitric oxide synthase (iNOS), cyclooxygenase-2 (COX-2), and serval pro-inflammatory cytokines (interleukin-1β (IL-1β), interleukin-6 (IL-6) and tumor necrosis factor α (TNF-α)). Mechanistic studies revealed that the anti-inflammatory effect of **MC** was associated with the activation of the mitogen activated protein kinases (MAPKs) (including p38, ERK and JNK) and nuclear factor-κB (NF-κB) pathways, especially reducing the nuclear translocation of NF-κB p65 subunit as revealed by nuclear separation experiment and confocal microscopy.

## 1. Introduction

Inflammation is a complex and highly orchestrated network of immunological, physiological, and behavioral events that takes place following exposure to various harmful stimuli from intrinsic and extrinsic sources including tissue injury, extreme temperature, stimulant, infection of pathogens, and metabolic disorder [1,2]. The primary function of inflammation is to eliminate underlying disturbance and restore tissue homeostasis [3]. However, inappropriate inflammatory response can become an inherent risk and a pivotal driver of countless major diseases such as rheumatoid arthritis, chronic inflammatory bowel diseases, type II diabetes mellitus, and psoriasis [1,4]. Overproduction of the multiple pro-inflammatory mediators, which are released by various immune cells, may contribute to the excessive inflammation in the body [5].

Releasing of inflammatory mediators during chronic inflammatory diseases is controlled by activation of intracellular signaling cascades. Toll-like receptors complex (TLRs) signal pathway is activated by the binding of LPS, which triggers downstream of nuclear factor κB (NF-κB) and the mitogen activated protein kinases (MAPKs) pathways [6,7]. NF-κB, representing a five-member collection of transcription factors, has been involved in both physiological and pathophysiological inflammatory responses [1]. Normally, NF-κB as an inactivated dimer composed of its p65 and p50 subunits stays in the cytoplasm [8]. Once the encountered inflammatory stimuli such as LPS, IκB is phosphorylated and degraded, NF-κB is released and transferred from the cytoplasm to the nucleus, which leads to the overexpression of several inflammatory mediators, including cyclooxygenase-2 (COX-2), inducible nitric oxide synthase (iNOS), tumor necrosis factor α (TNF-α), interleukin-1β (IL-1β), and interleukin-6 (IL-6) [7,9]. On the other hand, it has been recognized that MAPKs, consisting of p38 MAPK, extracellular signal-regulated kinase (ERK) and c-Jun N-terminal kinase (JNK), play a pivotal role in the pathogenesis of many inflammatory disorders [10]. The MAPKs signal pathway can activate the NF-κB pathway and work together with NF-κB pathway to aggravate the inflammatory diseases [10,11]. Therefore, the NF-κB pathway, as well as MAPKs, are emerging as potential therapeutic targets for the remedy of inflammatory diseases [12].

Natural products, especially polyphenolic compounds, have provided considerable value to chemical diversity for anti-inflammatory molecules over the past century [13,14,15]. *A. heterophyllus* is an important genus of Moraceae, which is known to be a rich resource of various beneficial polyphenols and is commonly used as a traditional medicine for malaria and fever in China [16]. *A. heterophyllus*, because of the nutritive value of its fruits and their biopharmaceutical activities, has attracted much research interest for decades. Extracts of this species, the chemical constituents of which are mainly flavonoids, especially flavones, isoflavones, chalcones, xanthones and prenylated stilbenes, have been reported to possess several positive effects, such as anti-inflammatory [17], antioxidant, anti-hyperglycemic, antiviral [18], and antimalarial activities [19]. However, the exact molecular mechanisms of those compounds are still unknown.

In this study, we used an LPS-induced RAW 264.7 cell as an inflammatory model to investigate the anti-inflammatory effects of multiple natural phenols isolated from *A. heterophyllus* and we discovered that only **MC** significantly restrained the production of NO and was in a positive relationship with the dosage, indicating that **MC** may possess an anti-inflammatory property. Additionally, our study further showed that **MC** may exert this anti-inflammatory activity by interfering with NF-κB and MAPKs pathways. Our findings implied that **MC** might represent a potential therapy for treatment of inflammatory diseases.

## 2. Results

### 2.1. Cytotoxicity on Macrophage

**Figure 1:**
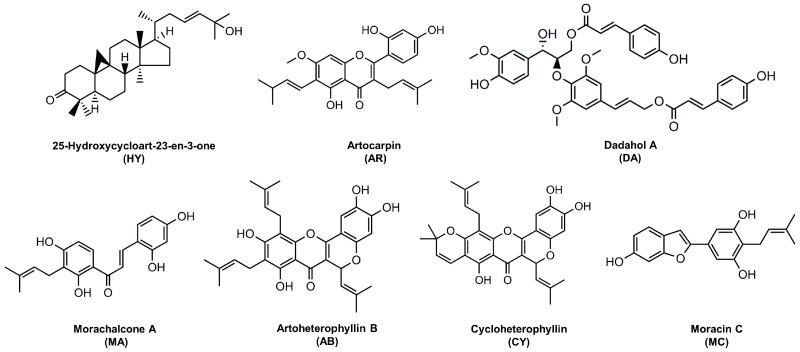
Chemical structure of compounds isolated from *A. heterophyllus*.

**Figure 2:**
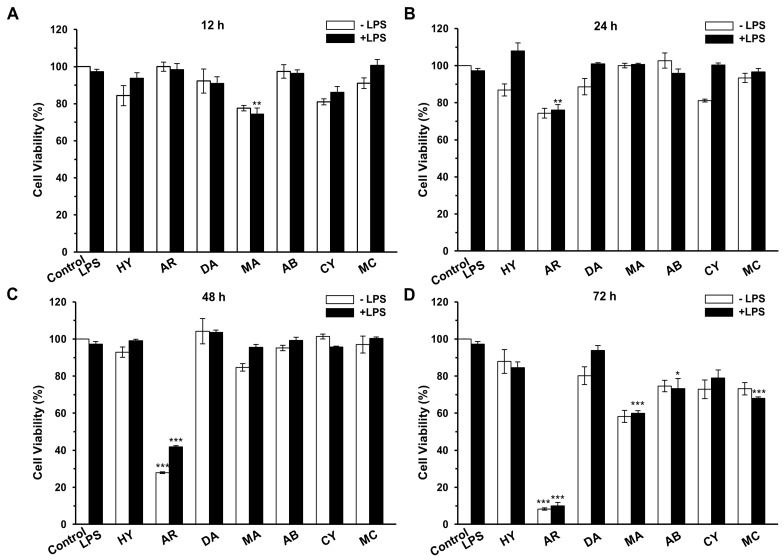
Effects of the seven compounds on the cell viability of RAW 264.7 cells. (**A**–**D**) RAW 264.7 cells incubated with 10 µM compounds for different times (12, 24, 48, and 72 h), with or without LPS treatment (1 µg/mL), respectively. The cell viability was measured using a MTT assay. Mean ± SEM, * *p* < 0.05, ** *p* < 0.01, *** *p* < 0.001.

Seven compounds isolated from *A. heterophyllus* were collected as shown in Figure 1. In addition to **HY** were the cycloartane derivatives [20]. All other compounds are polyphenolic compounds. Compound **AR**, **AB** and **CY** were proposed for the structure of prenylflavonoids [21,22,23]. Firstly, the cytotoxicity of all seven compounds were measured using RAW 264.7 cells. As shown in Figure 2, of these compounds, only **AR** exerted a significant cytotoxicity on RAW 264.7 cells after treatment at 48 or 72 h with or without LPS.

### 2.2. Effect of Natural Products on the Generation of NO

**Figure 3:**
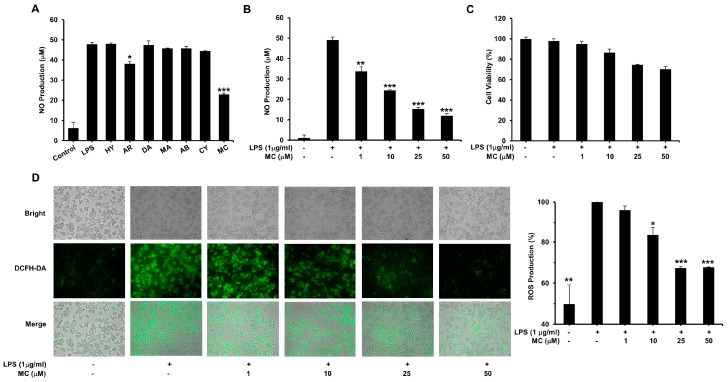
Effects of compounds on nitric oxide (NO) and reactive oxygen species (ROS) production in lipopolysaccharide (LPS)-activated RAW264.7 cells. (**A**,**B**) Cells were treated with 1 µg/mL LPS for 18 h. The nitrite concentration in the culture supernatant was detected to indicate NO production; (**C**) Cells incubated with 1, 10, 25 and 50 µM **MC** for 24 h. The cell viability was measured using a MTT assay; (**D**) Cells were treated with 1 µg/mL LPS for 1 h. The ROS content in the cell was detected using an oxidant-sensing fluorescent probe 2’,7’-dichlorofluorescein diacetate (DCFH-DA). **Left**: the images were observed using an inverted fluorescence microscope (Scale bar: 100 µm); **Right**: The quantitative data of fluorescent signal. Mean ± SEM, * *p* < 0.05, ** *p* < 0.01, *** *p* < 0.001.

To determine the effect of **HY**, **AR**, **DA**, **MA**, **AB**, **CY** and **MC** on NO generation, Griess reaction was performed. As shown in Figure 3A, the NO generation, measured as nitrite, rose observably from 6.2 µM of the vehicle to 47.8 µM when RAW 264.7 cells were treated with 1 µg/mL LPS. Among all tested compounds, only **MC** significantly suppressed the production of NO (IC_50_ = 7.70 µM) as shown in Figure 3B. Given that **MC** (50 µM) exhibited minor damage on the viability of RAW 264.7 cells in Figure 3C, the inhibitory action on NO production may not be attributable to cytotoxic effects. Taken together, **MC** was selected for subsequent anti-inflammatory activity and mechanism studies.

### 2.3. MC Reduces LPS-Induced ROS Production

We next determined whether **MC** could inhibit ROS production using the oxidation-sensitive probe DCFH-DA. The effect of **MC** on ROS generation induced by LPS in RAW 264.7 macrophages was demonstrated in Figure 3D. Treatment of cells with **MC** at 25 and 50 µM significantly inhibited LPS induced ROS generation, indicating that **MC** could significantly affect the ROS production in LPS-stimulated cells (Figure 3D), and act as an anti-oxidative compound to scavenge reactive radicals.

### 2.4. MC Inhibits LPS-Induced mRNA and Protein Expression of iNOS and COX-2

**Figure 4:**
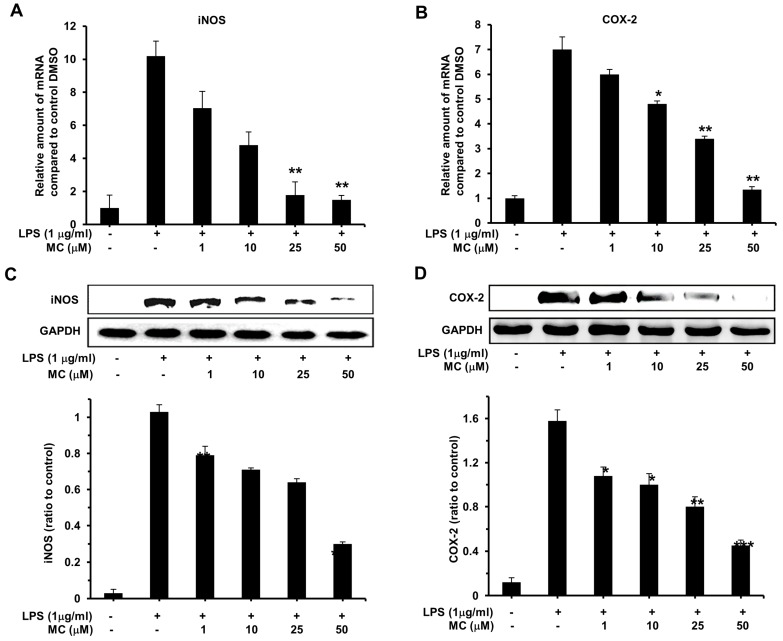
Effects of **MC** on mRNA and protein expression of iNOS and COX-2 in LPS-activated RAW 264.7 cells. Cells preincubated with 1, 10, 25 and 50 µM MC for 2 h were exposed to 1 µg/mL LPS for 24 h. Whole RNA was extracted for RT-PCR. Relative inducible nitric oxide synthase (iNOS) (**A**) and cyclooxygenase-2 (COX-2) (**B**) mRNA levels were calculated with reference to the LPS-treated group. The expressions of iNOS, COX-2, and GAPDH proteins were detected by Western blotting analysis. Relative iNOS (**C**) and COX-2 (**D**) protein levels were calculated with reference to the LPS-treated group. Mean ± SEM, * *p* < 0.05, ** *p* < 0.01, *** *p* < 0.001.

In order to further explore the molecular mechanism by which **MC** inhibits inflammatory reaction in response to LPS, we measured the mRNA and protein expression levels of iNOS and COX-2 through RT-PCR and western blotting assay, respectively. As shown in Figure 4, stimulation of RAW264.7 cells with 1 µg/mL LPS resulted in a dramatic increasing in mRNA and protein production level of iNOS and COX-2. Importantly, pretreatment with 1, 10, 25, and 50 µM **MC** respectively, could restrain the mRNA expression of both iNOS and COX-2 stimulated by LPS (Figure 4A,B), which revealed that **MC** may inhibit LPS-induced transcription of iNOS and COX-2. Additionally, the results of our Western blotting demonstrated that pretreatment with 10, 25, and 50 µM **MC** could affect the protein expression of both COX-2 and iNOS mediated by LPS (Figure 4C,D). In summary, these results demonstrated that **MC** was capable of reducing LPS stimulated mRNA and protein expression of both iNOS and COX-2.

### 2.5. MC Suppresses LPS-Induced Pro-Inflammatory Cytokines Expression

**Figure 5:**
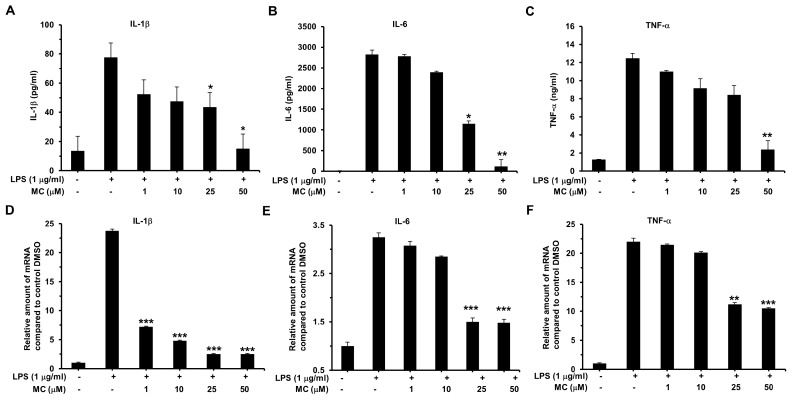
Effects of **MC** on pro-inflammatory cytokine productions in LPS-activated RAW 264.7 cells. Cells were treated with 1 µg/mL LPS for 24 h. IL-1β (**A**); IL-6 (**B**); and TNF-α (**C**) in the supernatant were detected by ELISA kits. Whole RNA was extracted for RT-PCR. Relative interleukin-1β (IL-1β) (**D**); interleukin-6 (IL-6) (**E**); and tumor necrosis factor α (TNF-α) (**F**) mRNA levels were calculated with reference to the LPS-treated group. Mean ± SEM, * *p* < 0.05, ** *p* < 0.01, *** *p* < 0.001.

The release of pro-inflammatory cytokines is one of the most important features of the LPS-induced inflammatory response, such as IL-1β, IL-6 and TNF-α. To clarify whether **MC** affect cytokine production, we conducted the ELISAs assay. As expected, LPS significantly induced the expression of IL-1β (5.7-fold, *p* < 0.005), IL-6 (253.0-fold, *p* < 0.005), and TNF-α (9.7-fold, *p* < 0.005). Pretreatment with 25 µM **MC** could reduce the secretion of IL-1β and IL-6 induced by LPS (Figure 5A,B) while LPS-mediated up-regulation of TNF-α was significantly reduced by 50 µM **MC** by 70.6% (Figure 5C).

We simultaneously determined the function of **MC** on the mRNA expression of IL-1β, IL-6 and TNF-α in LPS-induced RAW 264.7 cells by RT-PCR. As shown in Figure 5D–F, LPS up-regulated the mRNA expression of pro-inflammatory cytokines over that vehicle-treated cells while the mRNA levels of IL-1β, IL-6 and TNF-α were dramatically down-regulated in a dose-dependent manner when treated with **MC**.

### 2.6. MC Inhibits LPS-Induced TLR4 Expression and NF-κB Activation

Toll-like receptors (TLRs), particularly TLR4, play a crucial role in the well-known LPS-induced inflammatory pathways [24]. It has been reported TLR4 recognizes the lipid A component of LPS and induces the initiation of the activation of downstream signaling pathways like transcription factor NF-κB [25]. NF-κB is inactive when it was in the cytosol because of its inhibitor IκB, which was phosphorylated on LPS induction to release NF-κB, and IKK can phosphorylates IκB, as the upstream kinases of IκB in the NF-κB signal pathway [26].

**Figure 6:**
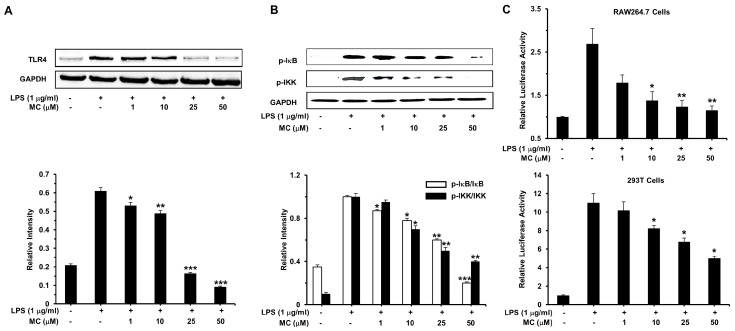
Effects of **MC** on LPS-induced activation of NF-κB pathway. (**A**) Western blotting analysis of the expression of TLR4 induced by LPS. Cells were treated with 1 µg/mL LPS for 24 h and total cell lysates were extracted and measured by Western blotting analysis; (**B**) Western blotting analysis of the phosphorylation of IκB and IKK expression induced by LPS. Cells were treated with 1 µg/mL LPS for 30 min and total cell lysates were extracted and measured by Western blotting analysis; (**C**) pNF-κB-luc were transfected into RAW 264.7 macrophages (**upper**) and HEK293T cells (**bottom**). Twenty-four hours after transfection, cells pretreated with **MC** for 2 h were treated with 1 µg/mL LPS for 6 h, the luciferase activity was determined. Mean ± SEM, * *p* < 0.05, ** *p* < 0.01, *** *p* < 0.001.

To further investigate the mechanism of MC-induced inhibition of iNOS, COX-2 and pro-inflammatory cytokines expression, in our study, western blotting was utilized to measure the expression of TLR4 and phosphorylation of IκB and IKK. As shown in Figure 6A, **MC** treatment for 24 h evidently blocked the LPS-mediated TLR4 expression, which may suggest that MC inhibits the expression of cell surface receptors TLR4 on RAW 264.7 macrophages. Figure 6B manifested that LPS markedly aroused the phosphorylation of IκB and IKK when exposing RAW 264.7 cells with 1 µg/mL LPS for 30 min. **MC** pretreating effectively suppressed these processes in a dose dependent manner. All of these results indicated that **MC** could effectively inhibit the activation of NF-κB and reduce TLR4 expression in LPS-induced RAW 264.7 macrophages (Figure 6A,B).

Furthermore, p65 subunits of NF-κB activated by LPS can be transferred into the nucleus and bind to the NF-κB binding site so as to enhance transcriptional activity of NF-κB [27]. Hence, pNF-κB-Luc reporter gene was used in the following study to determine whether **MC** affected the transcriptional activity of NF-κB. As we expected, NF-κB activity in LPS-stimulated RAW264.7 cells and 293T cells were 2.7-fold and 11.0-fold, respectively, as compared to the control group. Treatment with **MC** could repress the NF-κB-driven transcriptional activity both in RAW264.7 and 293T cells in a dose-dependent manner (Figure 6B).

**Figure 7:**
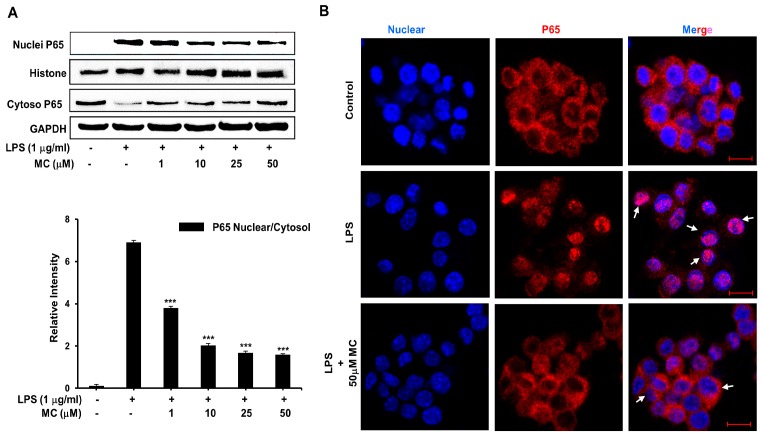
Effects of **MC** on LPS-induced translocation of NF-κB subunit p65. Cells preincubated with 50 µM **MC** for 2 h, were treated with 1 µg/mL LPS for 1 h. (**A**) Expression of NF-κB p65 protein was detected by Western blotting analysis; (**B**) The immunofluorescence analysis was performed with rabbit anti-NF-κB p65 antibody and an Alexa Fluor 555-conjugated anti-rabbit IgG antibody (**red**). Hoechst 33342 was used to label the nuclei (**blue**). The images were captured by confocal microscopy (Scale bar = 10 µm). The translocation of NF-κB p65 was marked by the arrows. The representative images from three independent experiments are shown here. Mean ± SEM, * *p* < 0.05, ** *p* < 0.01, *** *p* < 0.001.

In addition, given that releasing of NF-κB from the restraint of IκBα and the transsituation of NF-κB p65 into the nucleus is the most important process for the pro-inflammatory gene transcription, we determined the influence of **MC** on LPS-induced translocation of NF-κB p65 subunit. Western blotting revealed that **MC** dose-dependently attenuated p65 levels in nuclear fractions (Figure 7A). Confocal microscopy was used to disclose that in the control group without LPS stimulation, NF-κB p65 (red) mainly appeared in the cytoplasm. As shown by strong NF-κB p65 staining in the nucleus (Figure 7B), LPS treatment resulted in the translocation of p65 subunit from the cytoplasm to the nucleus (blue). The expression of p65 in the nucleus was distinctly inhibited by **MC** (50 µM). Our findings indicated that through preventing the LPS-induced nuclear translocation of p65, **MC** inhibited activation of NF-κB.

### 2.7. MC Inhibits LPS-Induced MAPKs Pathways Activation

**Figure 8:**
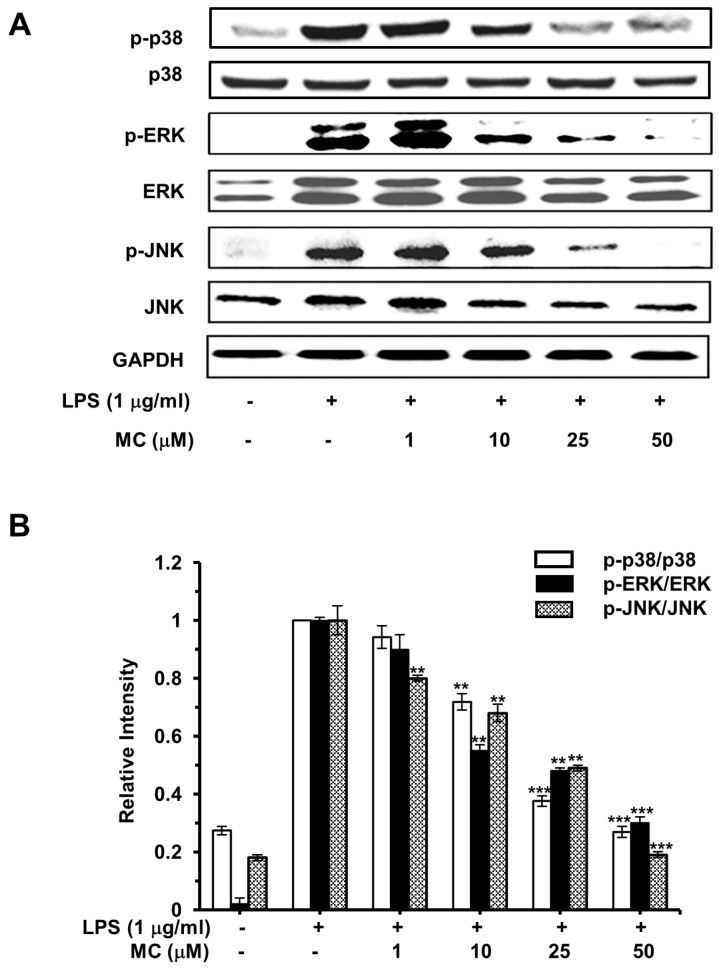
Effects of **MC** on the phosphorylation of MAPKs. (**A**) Cells preincubated with 1, 10, 25 and 50 µM **MC** for 2 h were exposed to 1 µg/mL LPS for 30 min, for the detection of phosphorylated and total p38, ERK and JNK, cell lysates were extracted and measured by Western blotting analysis; (**B**) The ratio of the phosphorylation and the non-phosphorylation of MAPKs. The representative Western blotting bands from three independent experiments are shown here. Mean ± SEM, ** *p* < 0.01, *** *p* < 0.001.

Accumulated evidence indicated that the MAPKs havebeen involved in the expression of a great deal of pro-inflammatory cytokines and the activation of NF-κB in response to LPS [28,29]. Therefore, we investigated the activation of the distinct MAPKs pathways to assess their potential involvement in the anti-inflammatory activities of **MC** by Western blotting analyses. As expected, the levels of activated p38, ERK and JNK were very low in the control group, but dramatically improved in cells following LPS stimulation. Pretreatment with **MC** markedly reduced phosphorylated p38, ERK and JNK levels in a dose-dependent manner as shown in Figure 8, implying that inhibitory effect of **MC** on activation of p38, ERK and JNK pathway was associated with the suppression of **MC** on LPS-initiated inflammatory responses.

## 3. Discussion

Chronic inflammation, a pivotal factor of pathogenesis of multiple diseases, has attracted wide concerns about public health and medical finance [30]. A continual increase in the incidence of inflammatory diseases has been observed worldwide, and great interest has been focused on identifying alternative approaches to regulate the inflammatory response. Natural products, especially dietary products, provide a promising future to the cure of chronic inflammatory diseases. In this study, we have screened the anti-inflammatory activity of seven natural products extracted from *A. heterophyllus*, and investigated the mechanism of action of **MC**. Our study demonstrated that **MC** markedly restrained inflammatory responses induced by LPS in RAW 264.7 cells through down-regulation of NF-κB and MAPKs pathways.

*A. heterophyllus*, (also known as the jackfruit tree), is popular in South-East Asian regions as a kind of tropical fruit. It has drawn much interest form researchers because of its potential pharmaceutical value as crude extracts and varied phytochemicals isolated from *A. heterophyllus* have been found to possess anti-inflammatory activity, which can be explained by the phenolic compounds including flavonoids, stilbenoids, and arylbenzofurons [31]. In this study, seven natural products **HY**, **AR**, **DA**, **MA**, **AB**, **CY** and **MC**, extracted from *A. heterophyllus* by BioBioPha (unpublished data), were collected to investigate their anti-inflammatory property using LPS-stimulated RAW264.7 macrophages. **AR**, **MA**, and **MC** have been reported to have inhibitory effects on NO production in LPS induced RAW 264.7 cells with the IC_50_ values of 18.7, 16.4 and 8.0 µM, respectively [23,32]. However, further investigation of the anti-inflammatory property and the specific mechanism of action have not been explained. In the present study, among all of the seven compounds, only **MC**, a phenolic compound, dramatically inhibited the overproduction of NO induced by LPS with an IC_50_ value of 7.70 µM with minor cytotoxicity (Figure 2) which was in accordance with the literature data (IC_50_ = 8.0 µM) [32]. Hence, we selected **MC**, as a potential agent, to further investigate the potential mechanism of the anti-inflammatory activity.

An abundance of researches have confirmed that NF-κB can regulate the expression of cytokines, growth factors, effector enzymes and genes, which is an indispensable process in the pathogenesis of inflammatory response [28,33]. As a consequence, to evaluate the specific mechanism of **MC** on NF-κB pathway, we detected the phosphorylation of IκB and IKK, which are two vital events of NF-κB activation. We found that LPS induction dramatically improved the phosphorylation levels of IκB and IKK, which was significantly blocked by pretreatment of **MC** (Figure 6A,B). Luciferase reporter assay was conducted to investigate the transcriptional activity and we found that **MC** significantly inhibited TNF-α-induced and LPS-induced NF-κB expression (Figure 6C). Moreover, as shown in Figure 7, LPS could markedly promote the translocation of p65 subunit of NF-κB and **MC** greatly blocked this effect of LPS. The TLR family are essential in the process of pathogen recognition and initiation of innate immunity. Moreover, signal transduction events induced by LPS are also involved with this receptor. We discovered that pretreatment of **MC** can clearly reduce the protein content of TLR4 in RAW264.7 macrophages. These findings suggested that **MC** could inhibit the expression of the membrane receptor TLR4, and suppress LPS-induced the activation of NF-κB pathway, especially the translocation of p65 subunit.

MAPKs are a highly conserved family of serine/threonine protein kinases that regulate basic physiological processes and cellular responses to extrinsic forces [34]. Much attention has been focused on development of MAPKs’ inhibitors since they can adjust the generation of various pro-inflammatory cytokines (for instance IL-1, IL-6, and TNF-α). Additionally, they play crucial roles in TLR, IL-1, IL-17 and TNF-α receptors-mediated signaling pathways, too. Moreover, LPS-induced iNOS and COX-2 production has been reported to be partly controlled by MAPKs [35]. Thus, we estimated the effect of **MC** on LPS-stimulated phosphorylation of p38, JNK and ERK to further explain the potential anti-inflammatory mechanism of **MC**. Figure 8 showed that **MC** inhibited the activation of p38, JNK and ERK in LPS-activated RAW 264.7, which suggests that p38, JNK and ERK are associated with the inhibition of LPS-mediated inflammation by **MC**.

Inhibition of NF-κB and MAPKs pathways has been proposed as the two major mechanisms to explain the restraint of LPS-initiated inflammatory cytokine production including the encoding cytokines of IL-1β, IL-6 and TNF-α, as well as inflammation associated enzymes including COX-2 and iNOS [36,37]. It has been reported that cytokines, especially TNF-α, can play a necessary synergistic role with LPS in induction of NO synthesis in macrophages. Suppression of inflammatory cytokines such as IL-1β, IL-6 and TNF-α are regarded as a treatment strategy on inflammatory diseases [38]. In our results, **MC** restrained overproduction of LPS induced NO related to the down-regulation of iNOS mRNA expression, as well as pro-inflammatory cytokines expression (IL-1β, IL-6 and TNF-α), in a dose dependent manner, which was in accordance with our results that **MC** inhibited the activation of NF-κB and MAPKs pathways.

In this study, we also measured the effect of **MC** on the generation of ROS using LPS-induced RAW264.7 macrophages and found that **MC** down-regulated the content of ROS in a dose-dependent manner. ROS is generated by inflammatory cells and accumulated in both allergic and non-allergic inflammation to kill the invading agents. However, overproduction of ROS in cells can also lead to inflammation and unavoidable tissue injury, which is crucial for the mechanism of inflammation [39,40,41]. Hence, suppression of ROS was helpful for the therapy of inflammatory diseases, especially for alleviation of tissue damages [42]. It has been reported that various pro-inflammatory mediators (IL-1β, IL-6, and TNF-α) regulated by NF-κB, can result in a massive generation of ROS. In turn, overproduction of ROS can lead to the activation of complex inflammatory regulating pathways (NF-κB pathways) [43]. Our study simply checked the effect of **MC** on the total production of ROS. We still need further studies to determine the detailed mechanism of the antioxidant activity as well as the relation between the antioxidant and anti-inflammatory activities of **MC**.

Our findings clearly demonstrate that **MC** modulates the inflammatory response of macrophages potently via signaling pathways involved with the NF-κB and MAPKs pathways. Considering that there are, unfortunately, often differences between the therapeutic effect in vitro and in vivo, the current evidence is limited to in vitro data, and more investigations are therefore needed to elucidate the in vivo relevance of our findings. In conclusion, we found **MC**, a natural phenolic product isolated from *A. heterophyllus*, exhibited a potent protective effect against LPS-induced inflammation response through reducing iNOS, COX-2, IL-1β, IL-6 and TNF-α protein production by suppressing the NF-κB and MAPKs activation. Hence, the findings from the current study support further investigations of **MC** for its anti-inflammatory potential.

## 4. Experimental Section

### 4.1. Materials and Reagents

All compounds isolated from *A. heterophyllus* were purchased from Yunnan BioBioPha (Kunming, China). Before use, all of the compounds were stored at −20 °C with a solvent of DMSO. LPS (from Escherichia coli, 055:B5), MTT, DCFH-DA and Griess reagent were obtained from Sigma-Aldrich (St. Louis, MO, USA). 5× mix RT master and AceQ qPCR SYBR Green Master Mix were purchased from TOYOBO (Wako, Osaka, Japan). TRIZOL reagent and lipofectamine 2000 were purchased from Invitrogen (Carlsbad, CA, USA). Primary antibody of iNOS and GAPDH were purchased from Santa Cruz (Santa Cruz, CA, USA). Antibodies against COX-2, p-IKK, p-IκB, ERK, p-ERK, JNK, p-JNK, p38, p-p38 were obtained from Bioworld (Nanjing, China). Primary antibodies of TLR4, NF-κB p65 were donated from Abway (Shanghai, China). Alexa Fluor 555-conjugated secondary anti-rabbit IgG antibody and Hoechst 33342 were obtained from Beyotime Biotechnology (Wuhan, China). Dual-Glo Luciferase Assay System was purchased from Promega (Madison, CA, USA).

### 4.2. Cell Culture

RAW 264.7 cells and HEK293T cells were purchased from the Institute of Biochemistry and Cell Biochemistry and Cell Biology (Shanghai, China) and were cultured in Dulbecco’s modified Eagle’s medium, supplemented with 2 mM l-glutamine, and 10% FBS, and maintained at 37 °C under 5% CO_2_ atmosphere.

### 4.3. Nitrite Assay

RAW 264.7 macrophages were seeded at a density of 1 × 10^5^ cells/well with 500 µL of DMEM plus 10% FBS in 24-well plates before incubation for 6 h. Then the cells were firstly incubated with tested compounds for 2 h and then stimulated with 1 µg/mL LPS for 18 h. Griess reaction was used to measure the NO concentration in the medium [33]. In details, 100 µL of cell culture supernant was added into equal volume of Griess reagent (0.1% naphthylethylenediamine dihydrochloride and 1% sulphanilamide in 5% phosphoric acid) in 96-well plates, and using a Synergy 2 Multi-Mode Microplate Reader (BioTek, Winooski, VT, USA), the absorbance at 540 nm was detected. By comparison with the OD_540_ values of a standard solution of sodium nitrite prepared in culture medium. Concentrations of nitrite in tested wells were calculated.

### 4.4. Cell Viability Assay

Cell viability was accessed by MTT assay. Detailed, RAW 264.7 cells were seeded in a 96-well plate (8 × 10^3^ per well) and incubated at 37 °C overnight. Then these RAW264.7 cells were treated with 10 µM of tested compounds or vehicle alone. After a serial time (12, 24, 48, and 72 h) of incubation at 37 °C, 20 µL of the MTT solution dissolved in PBS at 5 mg/mL was added to each well and incubated. Discarding the medium after 4 h and adding 100 µL DMSO into every well to dissolve the formazan precipitate [4], the absorption at the wavelength of 570 nm of the plates were measured on a Synergy 2 Multi-Mode Microplate Reader (BioTek, Winooski, VT, USA).

### 4.5. Measurement of the Content of ROS

The intracellular ROS generation was measured using a well-established probe, the oxidant-sensitive probe 2’,7’-dichlorofluorescein diacetate (DCFH-DA), to determine and quantify intracellular produced hydrogen peroxide [39,44]. RAW 264.7 cells were incubated with **MC** for 2 h and stimulated with LPS (1 µg/mL) for another 30 min. Then the cells were incubated with 10 µM DCFH-DA for 30 min, and washed twice with PBS solution. The fluorescence of DCF was observed using an inverted fluorescence microscope (Nikon, Tokyo, Japan) with a digital camera.

### 4.6. Cytokine Quantification

RAW 264.7 mouse macrophages were seeded in 24-well plates (at a density of 1 × 10^5^ cells per well) and incubated for 6 h. Before stimulated with 1 µg/mL LPS for 24 h, the cells were treated with **MC** for 2 h. The concentration of IL-1β, IL-6 and TNF-α in supernatants were determined using commercially available ELISA kits (Neobioscience Technology, Shenzhen, China) according to the manufacturer’s instructions.

### 4.7. RNA Extraction and Reverse Transcriptase (RT-PCR)

Total RNA were extracted using TRIZOL reagent in accordance to the manufacturer’s instruction. Then the total RNA was reverse-transcribed into cDNA using 5 × mix RT master (FSQ-201) under the following conditions: 37 °C for 15 min, 50 °C for 5 min, 98 °C for 5 min and 4 °C forever. The sense and antisense primers for iNOS were 5′-CCTGGTACGGGCATTGCT-3′ and 5′-GCTCATGCGGCCTCCTTT-3′, respectively. The sense and antisense primers for COX-2 were 5′-ATGCTCCTGCTTGAGTATGT-3′ and 5′-CACTACATCCTGACCCACTT-3′, respectively. The sense and antisense primers for TNF-α were 5′-CTGTAGCCCACGTCGTAGC-3′ and 5′-TTGAGATCCATGCCGTTG-3′, respectively. The sense and antisense primers for IL-6 were 5′-TGGAGTCACAGAAGAAGTGGCTAAG-3′ and 5′-TCTGACCACAGTGAGGAATGTCCAC-3′, respectively. The sense and antisense primers for IL-1β were 5′-ACTCCTTAGTCCTCGGCCA-3′ and 5′-CCATCAGAGGCAAGGAGGAA-3′, respectively. The sense and antisense primers for GAPDH were 5′-TGAAGCAGGCATCTGAGGG-3′ and 5′-CGAAGG TGGAAGAGTGGGAG-3′, respectively [45]. Q-PCR was performed using the AceQ qPCR SYBR Green Master Mix in accordance with the manufacturer’s protocol. GAPDH was used in this experiment to normalize the PCR amounts of every group.

### 4.8. Extraction of Total, Cytosol, and Nuclear Proteins

RAW 264.7 cells were pretreated with **MC** for 2 h. After induced by 1 µg/mL LPS for 60 min, the protein of cytosol and nuclear were harvested. Using a nuclear protein extraction kit (Beyotime Biotechnology, Wuhan, China), we isolated the cytoplasmic component from nuclear one form RAW 264.7 cells. All above procedures should be performed on ice. In addition, total proteins were also extracted from RAW 264.7 cells with RIPA buffer (Beyotime Biotechnology, Wuhan, China) and the bicinchoninic acid assay (BCA) was used to determine the protein concentration.

### 4.9. Western Blot Analysis

Protein samples after being quantified, were loaded, and separated by 10% sodium dodecylsulfate polyacrylamide gel electrophoresis (SDS-PAGE), then transferred to a PVDF membrane (GE Healthcare, Buckinghamshire, UK). After transfer, 5% BSA (in TBST buffer) was used to block the membrane at room temperature for 1 h [4]. Then the membranes were pre-incubated with primary antibody at 4 °C overnight and then incubated with secondary antibody following washing with TBST three times. Blots were visualized and quantified using Bio-Imaging System (BioTek, Winooski, VT, USA). The equivalent loading of proteins in each well was confirmed by GAPDH or histone control. The Image J software version 1.43u (National Institutes of Health, Bethesda, MD, USA) was used to quantified the gels.

### 4.10. NF-κB Luciferase Reporter Assay

The luciferase assay was performed as described with some modifications [46]. Mouse macrophages RAW 264.7 and HEK293T cells were used in our experiment and when we observed the confluence of the cells, transiently transfected was performed with the mouse pNF-κB-luc reporter plasmid using lipofectamine 2000 reagents. After another 6 h, we trypsinized the cells and equal numbers of cells (1.2 × 10^4^) were seeded in 96-well plates for 18 h. Different concentrations (1, 10, 25, and 50 µM, respectively) of **MC** or DMSO were added to the cells, and followed by stimulation with 1 µg/mL LPS for 6 h. Cells in each well were then washed twice with cold PBS and harvested in 150 µL of passive lysis buffer (0.5 M HEPES pH 7.8, 1% Triton N-101, 1 mM CaCl_2_, and 1 mM MgCl_2_) for luciferase assays (Promega, Madison, WI, USA). Transfection experiments were performed in triplicate and repeated at least three times.

### 4.11. Immunofluorescence Analysis

To examine nuclear location of NF-κB p65 subunit, we cultured RAW 264.7 cells onto glass coverslips in 6-well plates for 24 h. Before being treated with 1 µg/mL LPS for 1 h, the cells were pre-incubated with 50 µM of **MC** for 2 h. After that, we fixed the cells with 4.0% (*w*/*v*) paraformaldehyde for 15 min at room temperature. 0.5% (*v*/*v*) Triton X-100 was used to permeabilize the cells prior to blocking in 1% (*w*/*v*) goat serum in PBS for 1h. The cells were then treated with anti-NF-κB p65 antibody (1:400 in 1% goat serum/PBS) overnight at 4 °C following three washings with PBS. After Alexa Fluor 555-conjugated secondary anti-rabbit antibody (1:500) treatment for 2 h at room temperature, we stained the nuclei with 10 µg/mL Hoechst 33342 (Beyotime Biotechnology, Wuhan, China). The immunofluorescence analysis was performed using an A1R Confocal Microscope (Nikon, Tokyo, Japan).

### 4.12. Statistical Analysis

Data were expressed as the mean ± S.E.M from at least three independent experiments. One-way ANOVA and two-tailed Student’s *t*-test were performed. *p* values less than 0.05 were considered as statistically significant. All statistical tests were carried out using GraphPad Prism software (GraphPad Inc., San Diego, CA, USA).

## 5. Conclusions

In this study, we found that **MC**, a natural phenolic product isolated from *A. heterophyllus*, possess a potent protective effect against LPS-induced inflammatory responses in RAW264.7 macrophages, through suppressing the NF-κB and MAPKs activation. This may bring hope to the treatment of various inflammatory diseases.

## Abbreviations

AB	Artoheterophyllin B
AR	Artocarpin
COX-2	cyclooxygenase-2
CY	Cycloheterophyllin
DA	Dadahol A
ERK	extracellular signal-regulated kinase
FBS	fetal bovine serum
HY	25-Hydroxycycloart-23-en-3-one
IL-1β	interleukin-1β
IL-6	interleukin-6
iNOS	inducible nitric oxide synthase
JNK	c-Jun N-terminal kinase
LPS	lipopolysaccharide
MA	Morachalcone A
MAPKs	mitogen activated protein kinases
MC	Moracin C
NO	nitric oxide
NF-κB	Nuclear Factor-κB
ROS	reactive oxygen species
TLRs	Toll-like receptors
TNF-α	tumor necrosis factor α
